# Impact of Susceptibility on Plant Hormonal Composition during Clubroot Disease Development in Canola (*Brassica napus*)

**DOI:** 10.3390/plants12162899

**Published:** 2023-08-09

**Authors:** Charitha P. A. Jayasinghege, Jocelyn A. Ozga, Victor P. Manolii, Sheau-Fang Hwang, Stephen E. Strelkov

**Affiliations:** Plant BioSystems, Department of Agricultural, Food and Nutritional Science, University of Alberta, Edmonton, AB T6G 2P5, Canada; charitha.jayasinghege@agr.gc.ca (C.P.A.J.); vmanolii@ualberta.ca (V.P.M.); sheau.fang.hwang@ualberta.ca (S.-F.H.)

**Keywords:** *Plasmodiophora brassicae*, auxin, ethylene, salicylic acid, jasmonic acid, abscisic acid, root galls

## Abstract

Clubroot, caused by *Plasmodiophora brassicae*, is a soilborne disease of crucifers associated with the formation of large root galls. This root enlargement suggests modulation of plant hormonal networks by the pathogen, stimulating cell division and elongation and influencing host defense. We studied physiological changes in two *Brassica napus* cultivars, including plant hormone profiles—salicylic acid (SA), jasmonic acid (JA), abscisic acid (ABA), the auxin indole-3-acetic acid (IAA), and the ethylene precursor 1-aminocyclopropane-1-carboxylic acid (ACC)—along with their selected derivatives following inoculation with virulent and avirulent *P. brassicae* pathotypes. In susceptible plants, water uptake declined from the initial appearance of root galls by 21 days after inoculation, but did not have a significant effect on photosynthetic rate, stomatal conductance, or leaf chlorophyll levels. Nonetheless, a strong increase in ABA levels indicated that hormonal mechanisms were triggered to cope with water stress due to the declining water uptake. The free SA level in the roots increased strongly in resistant interactions, compared with a relatively minor increase during susceptible interactions. The ratio of conjugated SA to free SA was higher in susceptible interactions, indicating that resistant interactions are linked to the plant’s ability to maintain higher levels of bioactive free SA. In contrast, JA and its biologically active form JA-Ile declined up to 7-fold in susceptible interactions, while they were maintained during resistant interactions. The ACC level increased in the roots of inoculated plants by 21 days, irrespective of clubroot susceptibility, indicating a role of ethylene in response to pathogen interactions that is independent of disease severity. IAA levels at early and later infection stages were lower only in susceptible plants, suggesting a modulation of auxin homeostasis by the pathogen relative to the host defense system.

## 1. Introduction

Clubroot of crucifers is a widespread and highly destructive root disease that affects plants in the *Brassicaceae* family, including the genus *Brassica*, which includes many economically important crops [1]. Canola (*Brassica napus* L.), also known as oilseed rape, is one of the most valuable oil crops in the world and is particularly threatened by this disease. Clubroot is caused by the protist *Plasmodiophora brassicae* Wor., an obligate soilborne parasite. Infected plants produce root galls that suppress normal water and nutrient absorption, resulting in stunted aboveground growth and low, poor-quality yields [2]. Currently, the primary approach for disease control in canola is to cultivate varieties bred for clubroot resistance. However, new pathotypes that can overcome resistance are constantly emerging, making disease management a continuing challenge [3,4]. Therefore, the long-term sustainability of canola production requires an integrated approach to clubroot disease management that includes resistant cultivars, appropriate cultural practices, chemical controls, and other preventive measures [5,6]. Manipulating the levels or actions of plant hormones implicated in root gall development, plant defense, and stress responses could provide an additional tool for controlling the disease in such an integrated approach [7]. However, the current understanding of the plant’s hormonal regulation and response during clubroot disease development remains incomplete. Developing any hormone-based approach requires a clear understanding of the hormonal regulation of the disease development.

The clubroot pathogen survives in the soil as long-lived resting spores. Germinated spores produce free-swimming primary zoospores that infect host root hairs. The infection of the root hairs, known as primary infection, does not result in root galls. Instead, the pathogen divides in the root hairs to form secondary zoospores, which are released back into the soil. These secondary zoospores then return to the roots, initiating a secondary cycle and infecting the cortex, stele, and hypocotyl. The extensive cell division and enlargement that occur during secondary infection produce root galls in which the pathogen multiplies, creating a myriad of new resting spores [8,9]. Plants of *B. napus* infected with this localized root parasite can produce several grams of root galls, with each gram releasing about 10 billion resting spores back into the soil as the galls decompose [10]. The resting spores can survive up to 15 years, serving as inoculum to infect and produce another generation of resting spores [11]. As a result, once a field becomes infested with *P. brassicae,* the pathogen is difficult to eradicate.

The first clubroot-resistant *B. napus* cultivar was introduced into the Canadian market in 2009, soon followed by many others. While these clubroot-resistant cultivars were not entirely immune, they showed excellent resistance to the *P. brassicae* pathotypes prevalent in western Canada, where most Canadian canola is grown [12]. However, within four years of the introduction of the resistance trait, ‘new’ pathotypes capable of overcoming clubroot resistance began to emerge that can cause severe clubroot in previously resistant cultivars. These newer pathotypes now predominate in many infested fields [3]. The genetic resistance to *P. brassicae* in many Brassica species appears to rely on one or a few major resistance genes [13,14,15]. The rapid emergence of new pathotypes capable of overcoming major gene resistance highlights the importance of developing new canola varieties that confer resistance through other genetic and physiological mechanisms. Such resistance mechanisms may improve the durability of clubroot resistance when combined with major gene resistance. One such approach to diversify resistance is through the identification of quantitative trait loci (QTLs) that contribute to *P. brassicae* resistance [16,17,18]. Understanding how the pathogen manipulates the hormonal profiles of the host to overcome its defense mechanisms and stimulate root gall development, and identifying hormone-based mechanisms that facilitate host defense, are also areas of interest, as they may provide options to modify plant hormonal actions for improved disease resistance.

Multiple factors can modulate plant hormonal profiles in infected plants. First, root gall development is likely facilitated by the growth-promoting hormones of the host. Among these, auxin, given its role in cell division and elongation, is long considered a possible player [19,20]. Cytokinin, a major stimulator of plant cell division, is another potential contributor. Interestingly, the presence of two putative isopentenyltransferase homologs in the *P. brassicae* genome, which regulate cytokinin biosynthesis and their high expression during gall formation, suggests that the pathogen itself is capable of producing some cytokinins. The impact of any pathogen-derived cytokinin on the host cytokinin profile, however, appears to be minimal [21]. In addition, there are indications that brassinosteroids may also play a positive role in clubroot disease development [22].

The second contributor affecting hormonal profiles is the defense response of the infected plants. Salicylic acid (SA) and jasmonic acid (JA) are the major plant hormones that regulate defense mechanisms. SA, the primary defense hormone against biotrophic pathogens, accumulates in both local and distal plant tissues to activate defense responses at and beyond the site of infection, leading to systemic acquired resistance [23]. Jasmonic acid and its derivatives, collectively known as jasmonates (JAs), play defense roles against necrotrophic pathogens. In addition, some evidence suggests they may also play a role in plant responses to certain biotrophs [24]. Various studies in plants of the *Brassicaceae* family infected with *P. brassicae* that involved hormonal treatments, hormonal profiling, and mutant analyses suggest regulatory roles of both JA and SA in clubroot disease development [25,26]. The gaseous plant hormone ethylene, which plays a role in diverse processes associated with plant development, is also known to contribute to plant defense in multiple ways, mainly through interactions with JA and SA signaling pathways [27]. To date, there is no evidence indicating a direct involvement of ethylene in clubroot disease; however, analysis of ethylene signaling mutants in Arabidopsis has led to the conclusion that ethylene signaling is important in restricting disease development [28]. A third way in which plant hormone profiles may change in infected plants is in response to abiotic stress responses associated with root gall development. Abscisic acid (ABA), a central regulator of drought responses, is likely to increase in diseased plants due to the limited free roots available for water uptake [29].

Despite a long-held interest in understanding the roles of plant hormones in clubroot development, their roles are still not clear due to the complexities of plant hormonal function in this process. For example, each hormone may have overlapping roles in disease development, plant defense, and stress responses associated with clubroot, as well as their normal roles in plant growth and development. The changes in hormone profiles may also vary based on the host species, cultivar, *P. brassicae* pathotype, stage of disease progression, and/or environmental factors. It is also likely that the pathogen may act to alter the profiles of certain hormones, further complicating our understanding. Therefore, broader evaluations of hormonal changes in different plant–pathogen combinations that show susceptible and resistant interactions are essential to obtain a clearer picture of pathogenesis. In this study, the modulation of the profiles of the defense hormones SA and JA was evaluated in canola hybrids showing susceptible or resistant interactions with two *P. brassicae* pathotypes (one pathotype causing disease in only one cultivar and the other causing disease in both cultivars). We also evaluated physiological changes in the plant in response to disease development and the modulation of root ABA levels in parallel with changes in plant water uptake. In addition, the levels of the auxin indole-3-acetic acid (IAA) and the ethylene precursor 1-aminocyclopropane-1-carboxylic acid (ACC) level were evaluated from early infection to the rapid root gall development stages. Our study contributes toward understanding hormonal modifications in *P. brassicae*-infected plants in response to susceptible and resistant interactions in canola at different stages of disease development.

## 2. Results and Discussion

### 2.1. Clubroot Susceptibility and Physiological Changes in Plants during Disease Development

**Figure 1:**
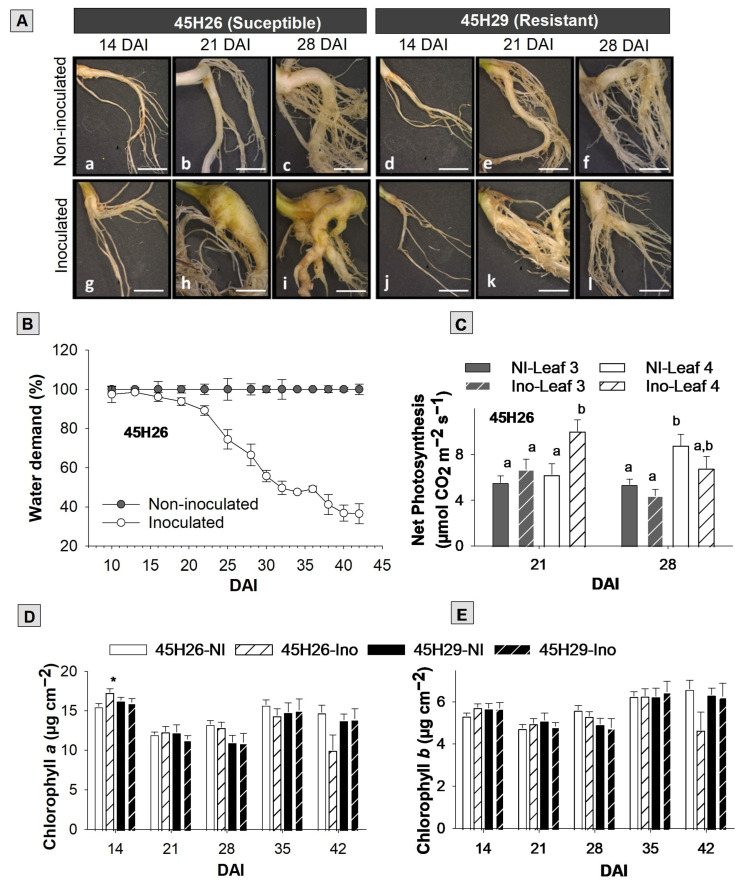
Physiological changes in canola plants in response to clubroot disease development. The susceptible canola cultivar 45H26 and resistant cultivar 45H29 were inoculated with *Plasmodiophora brassicae* pathotype 3H. (**A**) Representative roots from susceptible and resistant plants shown at 14, 21, and 28 days after inoculation (DAI). (**B**) Percentage water demand of inoculated 45H26 plants relative to non-inoculated controls. (**C**) Net photosynthesis in the third and fourth true leaves of inoculated (Ino) and non-inoculated (NI) plants of 45H26. (**D**) Chlorophyll *a* and (**E**) chlorophyll *b* levels in the leaves of the two host cultivars with and without *P. brassicae* inoculation. Data are means ± SD (in (**B**), where n = 3) or SE (in (**C**), where n = 12, and in (**D**,**E**), where n = 8–9). In (**C**), different letters denote significant differences within DAI by one-way ANOVA followed by the Holm–Sidak post-hoc test (*p* ≤ 0.05). In (**D**,**E**), an asterisk (*) denotes significant differences from that of the non-inoculated control within the cultivar and DAI (two-tailed Student’s *t*-test, *p* ≤ 0.05).

Two canola cultivars (45H26 and 45H29) with different responses (either susceptible or resistant) to *P. brassicae* pathotypes 3A and 3H were used in this study. Pathotype 3H was predominant in the Canadian province of Alberta prior to the introduction of clubroot-resistant hosts, but is also found elsewhere. Pathotype 3A emerged following the widespread cultivation of clubroot-resistant canola crops, and is capable of causing severe clubroot even in many 3H-resistant cultivars [3]. The cultivar 45H26 is susceptible to both pathotypes 3A and 3H, while 45H29 is susceptible to pathotype 3A but resistant to pathotype 3H [3]. As expected, when the canola cultivar 45H26 was inoculated with pathotype 3H, it produced distinct root galls by 21 days after inoculation (DAI). By 28 DAI, the galls were enlarged and had spread to the lateral roots, greatly reducing the portion of roots without any galls or swelling. In contrast, plants of the resistant cultivar 45H29 mostly produced no galls with only a few plants developing some small galls or swellings on the lateral roots (Figure 1A). When the disease symptoms were rated to calculate an index of disease (ID) based on the spread and severity of these root galls at 42 DAI, the 45H26 (susceptible) plants had an average ID (±SD) of 99.3 ± 0.9%, indicating severe galling in all plants, while the 3H-resistant cultivar 45H29 had an average ID of 13.4 ± 4.8%, indicating minor galling in a few plants. Inoculation of plants with pathotype 3A caused severe galling (ID of 100%) in both cultivars. The clubroot pathogen can infect root hairs irrespective of host susceptibility, including some non-host species. However, secondary infections that cause root galls only occur in host species. Even if a host is resistant to a given *P. brassicae* pathotype, some small galls may be visible under high inoculum pressure [30,31,32]. Therefore, severe clubroot symptoms on 45H26 following inoculation with either pathotype, and on 45H29 following inoculation with pathotype 3A, were expected, as were mild symptoms (a few small galls) on 45H29 inoculated with pathotype 3H.

In association with root gall formation, infected susceptible plants showed a definite decline in water uptake by 21 DAI, which continued until the end of the evaluation period at 42 DAI (Figure 1B). Both obstructed root water uptake by the developing root galls, and reduced plant water requirements due to suppressed shoot growth may have contributed to the observed decline. As noticeable root galls appeared around 3 weeks following inoculation and galls enlarged rapidly over the subsequent 2 weeks, the timeline of declining water uptake correlates well with that of root gall formation. As such, the percentage of water uptake could be used as a simple, real-time indicator to estimate clubroot disease progression in canola.

To understand the impact of root gall formation on photosynthesis, we evaluated photosynthetic rates in the leaf attached to nodes 3 and 4 in cultivar 45H26 inoculated with pathotype 3H at 21 and 28 DAI. Despite the appearance of root galls and the declining water uptake at this stage, net photosynthesis was not affected significantly in diseased plants, except for a slight increase in leaf position 4 at 21 DAI (Figure 1C). An earlier study in Chinese cabbage (*Brassica rapa* L. ssp. *pekinensis*) also reported an increase in photosynthesis as a result of clubroot disease [33]. Generally, high sink strength due to the high rate of sugar utilization resulting from processes such as cell division and growth in plant organs stimulates photosynthesis, and low sink activities result in feedback downregulation of photosynthesis [34]. Accordingly, the observed increase in photosynthesis suggests that during the initiation phase of rapid gall enlargement, plants are attempting to boost their photosynthetic capacity to cope with the increasing demand for photoassimilates in the roots. However, resource limitations likely affect the maintenance of high photosynthesis rates, leading to restricted allocations of photoassimilates for normal plant development, resulting in stunted aboveground growth.

Consistent with the observed maintenance of photosynthesis at or above the level of non-inoculated controls, stomatal conductance, transpiration, and photosynthetic water use efficiency also showed no clear difference in inoculated plants compared with non-inoculated controls at 21 and 28 DAI (Figure S1). As drought-stressed plants are expected to show a decline in stomatal conductance and photosynthesis [35], the absence of a significant difference in those measurements between healthy and infected plants during 21–28 DAI suggests that the decline in water uptake is not causing a substantial water deficit in infected plants. Therefore, the reduced water uptake during this period could be due primarily to reduced demand associated with stunted plant growth, rather than interference in uptake caused by the root galls.

Next, we examined leaf chlorophyll concentrations in 45H26 (susceptible) and 45H29 (resistant) plants inoculated with pathotype 3H. Chlorophyll *a* and *b* levels were not affected by pathogen inoculation in either cultivar when measured from 14 to 42 DAI (Figure 1D,E). These observations are consistent with previous observations in Chinese cabbage and Arabidopsis [33], as well as similar photosynthesis rates observed between infected and non-infected plants. As root gall development likely impacts plant nutrient uptake and contributes to deficiencies of multiple nutrients, particularly nitrogen that affects chlorophyll production [36], it is reasonable to expect an impact on chlorophyll content in susceptible plants. However, symptoms of nitrogen deficiency usually appear in mature leaves, and those leaves may have dropped prematurely in diseased plants. Therefore, the leaf selection criteria used here for chlorophyll analysis (leaves attached to the third node at 28 DAI and the second remaining leaf from the bottom at 35 and 42 DAI) may have masked any impact of clubroot development on leaf chlorophyll levels.

### 2.2. ABA Level Increases in Susceptible Plants

**Figure 2:**
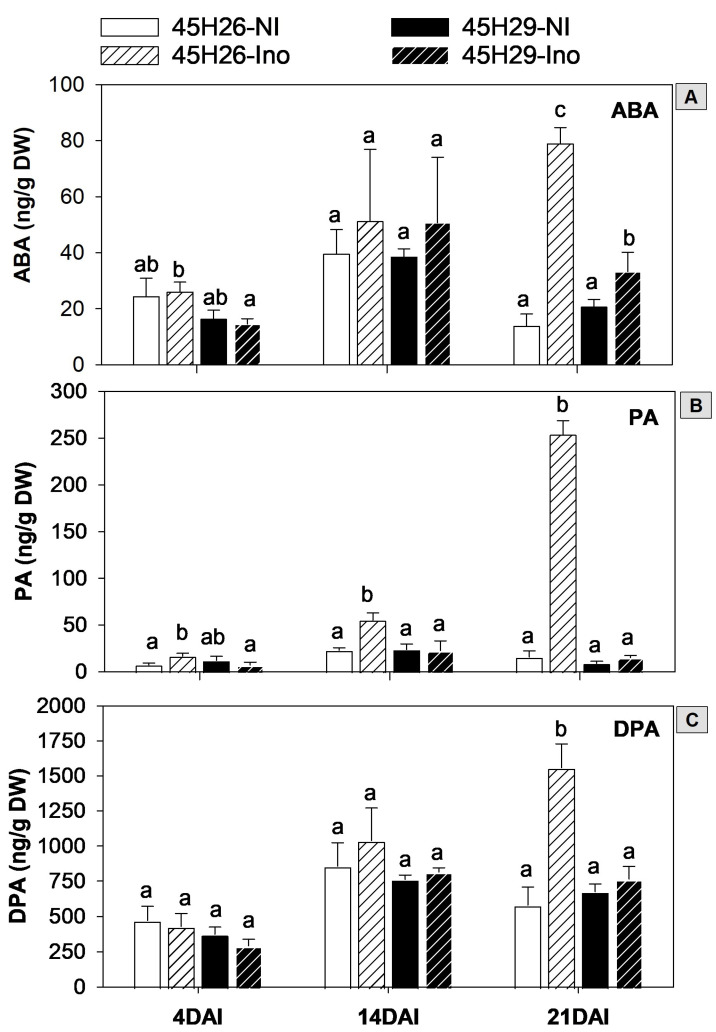
Effect of *Plasmodiophora brassicae* inoculation on abscisic acid (ABA), phaseic acid (PA) and dihydrophaseic acid (DPA) levels in the roots of canola plants. Modulation of ABA (**A**) and its metabolites PA (**B**) and DPA (**C**) levels in the roots of the canola cultivars 45H26 (susceptible) and 45H29 (resistant) at 4, 14, and 21 days after inoculation (DAI) with *P. brassicae* pathotype 3H (Ino) and in non-inoculated (NI) controls. Different letters denote significant differences within DAI as determined by one-way ANOVA followed by the Holm–Sidak post-hoc test (*p*  ≤  0.05). Data are means ± SD, n = 3 (with each sample composed of 60–78 plants).

In the absence of *P. brassicae* infection, the root ABA level did not differ between the host cultivars when evaluated at 4, 14, and 21 DAI. Inoculation with pathotype 3H also had no impact on root ABA levels within each cultivar at 4 and 14 DAI. By 21 DAI, however, ABA levels had increased by approximately 5.7-fold in 45H26 (susceptible) and 1.6-fold in 45H29 (resistant) inoculated plants compared with their respective non-inoculated controls (Figure 2A). Similarly, a study in Chinese cabbage also showed no significant change in ABA levels early in disease development (at 6 and 13 DAI), but a significant increase at 21 DAI [29]. Another study in *B. napus* showed no significant increase of ABA level in the roots of infected plants until 49 DAI [25].

The level of ABA in plants is regulated mainly through the balance between ABA biosynthesis and catabolism. Catabolism occurs through hydrolyzation or conjugation via several different routes. Among those, 8′-hydroxylation of ABA is a common route of metabolism, which produces phaseic acid (PA), an intermediate product that still has some ABA-like activity. Further metabolism of PA generates biologically inactive dihydrophaseic acid (DPA) [37]. The two host cultivars had similar root PA and DPA levels in the absence of pathogen infection at 4, 14, and 21 DAI (Figure 2B). Inoculation with *P. brassicae* pathotype 3H increased PA levels in the susceptible (45H26) plants at all the time-points tested. The increase in inoculated 45H26 plants was most prominent at 21 DAI, where the PA level was 16-fold higher than in the non-inoculated controls. PA levels did not increase in the resistant cultivar 45H29 at any of the time-points evaluated. Pathogen inoculation also increased root DPA levels in the susceptible plants by approximately 2.7-fold at 21 DAI, but not in the resistant plants (Figure 2C).

The increase in the levels of ABA and its metabolites PA and DPA in the susceptible inoculated plants by 21 DAI (Figure 2) was associated with the rapid expansion of root galls and decreased water uptake (Figure 1A,C) at this stage. ABA is a major regulator of drought stress responses. Increased ABA levels, along with those of PA and DPA in the susceptible cultivar, served as a marker for clubroot-induced stress by 21 DAI in these plants. The slight but significant increase in the ABA level in the resistant inoculated plants at 21 DAI also suggests that the presence of the pathogen stimulated stress responses in this cultivar. There is evidence that ABA may play a role in defense responses. While ABA may promote disease resistance in some host–pathogen interactions, it increases susceptibility in others [38,39]. In particular, ABA may antagonistically affect interactions with the plant defense hormone SA, which facilitates the infection process of certain pathogens by alleviating SA-mediated defense responses. Some pathogens even appear to produce ABA, stimulate plant ABA biosynthesis, or decrease ABA catabolism, likely as a way to dampen plant defense responses [38,39]. Additionally, abiotic stresses like drought are known to weaken biotic stress resistance in plants [39]. Consequently, elevated levels of ABA can lead to stomatal closure, impairing normal plant development, which possibly facilitates the progression of pathogens in infected plants. Therefore, even if *P. brassicae* has no direct role in modulating the host ABA level, the increased level of ABA during root gall development may inadvertently assist disease progression.

### 2.3. Salicylic Acid Level Is High during Resistant Interactions

**Figure 3:**
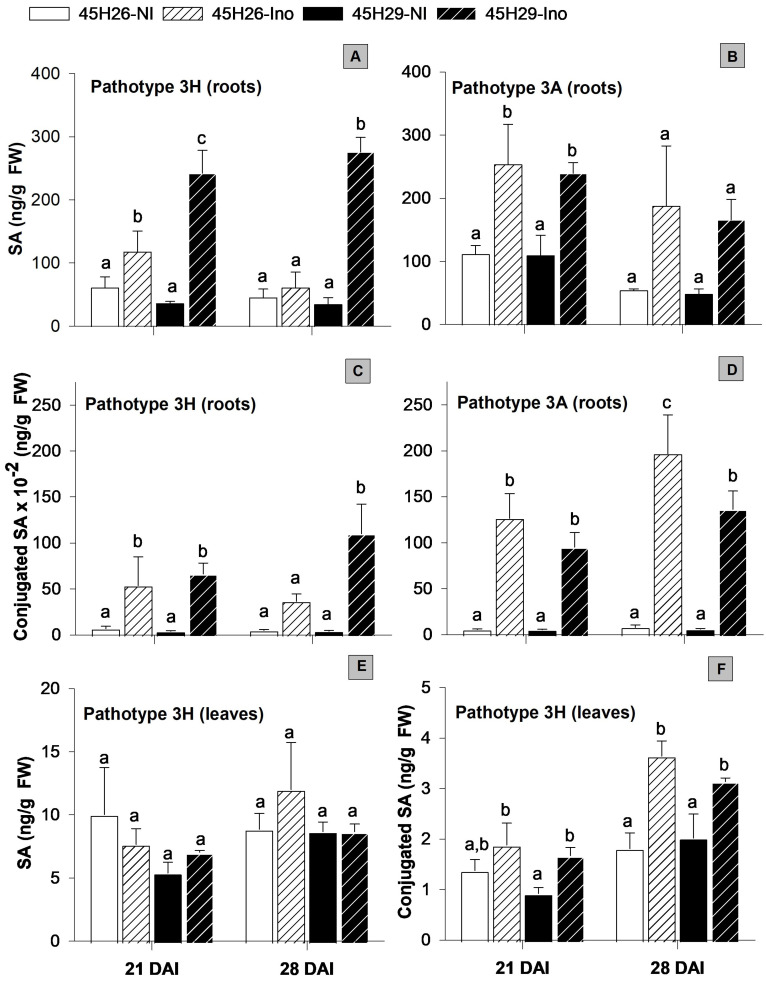
Effect of *Plasmodiophora brassicae* inoculation on salicylic acid (SA) levels in canola root and leaf tissues at 21 and 28 days after inoculation (DAI). Free (**A**,**B**) and conjugated (**C**,**D**) SA levels in the roots of cultivars 45H26 and 45H29 cultivars inoculated (Ino) with *P. brassicae* pathotypes 3H (virulent on 45H26 and avirulent on 45H29) or 3A (virulent on both cultivars) and in non-inoculated (NI) controls. Free (**E**) and conjugated (**F**) SA levels in the leaves of 45H26 and 45H29 plants inoculated or not inoculated with pathotype 3H. Data are means ± SD, n = 3 (with each sample composed of 24–30 plants). Different letters denote significant differences within DAI as determined by one-way ANOVA followed by the Holm–Sidak post-hoc test (*p* ≤ 0.05).

Salicylic acid (SA) was measured in the roots and leaves, as SA can accumulate beyond the site of infection as a mechanism of systemic acquired resistance. There were no significant differences in SA levels between non-inoculated 45H26 and 45H29 plants when evaluated in the leaves and roots at 21 and 28 days after inoculation (DAI). When inoculated with pathotype 3H, however, the free SA level in the roots of 45H29 (resistant) increased by approximately 7 to 8-fold at 21 and 28 DAI compared with the non-inoculated controls. The SA level also increased in the roots of 45H26 (susceptible) plants, but this increase was only 2-fold at 21 DAI and no change was observed at 28 DAI (Figure 3A). Foliar SA levels were not altered in either cultivar in response to pathotype 3H inoculation at the two time-points examined (Figure 3E).

In addition to its free form, plants convert SA into various derivatives, including glucose and amino acid conjugates and methylated SA for fine-tuning activity. The glycosylated and methylated SA are generally considered biologically inactive with respect to the stimulation of the plant defense responses but can be converted back to active SA [40]. In the absence of pathogen inoculation, total SA conjugate levels detected by acidic hydrolysis of SA extracts [41] showed no difference in the roots between the two host cultivars at 21 and 28 DAI. Inoculation with pathotype 3H increased conjugated SA levels in the roots of both cultivars at 21 DAI, but only in the resistant cultivar 45H29 at 28 DAI (Figure 3C). When the ratio of the SA conjugate to SA was compared, the roots of susceptible plants (45H26) inoculated with pathotype 3H had an SA conjugate to SA ratio of approximately 4- and 5.5-fold at 21 and 28 DAI compared with non-inoculated controls, respectively (Figure S2A). This ratio was lower in the resistant inoculated plants (45H29), and was 2.5- and 3-fold at 21 and 28 DAI, respectively. Inoculation with the pathogen also increased conjugated SA levels in the leaves up to approximately 2-fold; however, this change was not cultivar-dependent (Figure 3F).

Free SA and SA-conjugate levels in response to *P. brassicae* inoculation with pathotype 3A, which causes severe galling in both cultivars, followed trends similar to those observed with pathotype 3H during susceptible interactions. Both cultivars showed only a limited increase in SA levels at 21 DAI (2-fold) and upward trends but no significant increases at 28 DAI (Figure 3B). The ratio of SA conjugate to free SA was also greater in both cultivars inoculated with 3A (approximately 10- and 8-fold in 45H26 and 8- and 6.5-fold in 45H29 at 21 and 28 DAI, respectively; Figure S2B). Additionally, when inoculated with pathotype 3A, both cultivars exhibited a greater SA conjugate to free SA ratio compared with inoculation with pathotype 3H at 28 DAI (Figure S3).

The observed increase in SA in both cultivars with both pathotypes at 21 DAI suggests that *P. brassicae* infection triggers an accumulation of SA in the roots independent of host susceptibility (Figure 3). However, the resistant interaction between pathotype 3H and the cultivar 45H29 was associated with a stronger (at 21 DAI) and more persistent increase (at 28 DAI) in SA levels than that observed during susceptible interactions in 45H26. Similar trends have been reported previously in the *B. napus* cultivars Hornet and SY Alister. Hornet is highly susceptible to *P. brassicae* pathotype 6, as defined by the differentials of Williams, 1966 [42], while SY Alister shows partial resistance. In the roots of inoculated-resistant plants, SA levels increased at 35, 42, and 49 DAI compared with the controls. There was no change in the SA level in the susceptible cultivar except for an increase at 49 DAI [25]. Similar results were also reported in the Arabidopsis accessions Col-0 and Bur-0, which showed susceptible and partially resistant interactions with *P. brassicae* isolate e3, respectively. In resistant plants, pathogen inoculation activated host defense responses and an increased SA level was detected at 14 DAI. There was no such change in SA levels or signaling in the susceptible accession [26]. In addition to higher SA levels during resistant interactions, SA application has also been shown to reduce disease severity in Arabidopsis [26] and *B. napus* [25]. These findings provide evidence supporting an important and positive role of SA in clubroot resistance.

A role of SA in clubroot defense was also confirmed by changes in gene expression related to SA biosynthesis, translocation, and accumulation and signaling. For example, a stronger stimulation of SA-mediated defense genes has been reported during resistant interactions in the rutabaga (*B. napus* spp. *napobrassica*) cv. Laurentian compared with susceptible interactions in the oilseed rape (*B. napus*) cv. Brutor following inoculation with *P. brassicae* pathotype 5X [43]. These genes included *CALMODULIN-BINDING PROTEIN 60g* (*CBP60g*) and *SYSTEMIC ACQUIRED RESISTANCE DEFICIENT 1* (*SARD1*) that play roles in SA biosynthesis and other defence responses; *ISOCHORISMATE SYNTHASE 2* (*ICS2*), associated with SA biosynthesis; *ENHANCED DISEASE SUSCEPTIBILITY 5* (*EDS5*), associated with the translocation of isochorismate produced in the chloroplast into the cytoplasm for SA production [44]; *AVRPPHB SUSCEPTIBLE 3 (PBS3)* that catalyzes the conjugation of glutamate to isochorismate to produce isochorismate-9-glutamate, which spontaneously decomposes into SA in the cytoplasm [44]; and *NITROGEN LIMITATION ADAPTION* (*NLA*), which regulates SA-mediated immune responses [43]. Similarly, an inbred *B. napus* line with stronger clubroot resistance than its parents expressed higher levels of the SA receptor NON-EXPRESSOR OF PATHOGENESIS-RELATED GENES1 (NPR1, also known as NIM1) and SA-inducible [45] NIM1-INTERACTING (NIMIN) protein-coding genes [46].

The higher levels of SA observed during resistant compared with susceptible interactions with pathotype 3H could reflect the robust activation of defense mechanisms in the resistant plants. Furthermore, evidence suggests that *P. brassicae* can reduce host resistance by modulating SA levels in the plant. The clubroot pathogen possesses a methyl transferase gene (*PbBSMT*) that shows distant homology to SABATH family methyltransferases in plants [47]. Functional characterization of *PbBSMT* both in vitro and in transgenic overexpression lines has shown that PbBMST effectively methylates SA and that plants overexpressing *PbBSMT* are more susceptible to *P. brassica* infection. Therefore, *P. brassicae* is speculated to have the ability to methylate host SA to reduce SA availability as a way of evading SA-mediated plant defenses [47,48,49]. Further supporting this possibility, a transcriptomic analysis comparing symptomless roots and root galls within the same *P. brassicae*-infected kohlrabi (*Brassica oleracea* var. *gongylodes*) host showed that *PbBSMT* is one of the highest expressed genes in galled tissues. On the other hand, symptomless root tissues showed an upregulated expression of SA-mediated defense response genes compared with galled tissues, suggesting that *PbBSMT* may be causing a local reduction in SA, facilitating root gall development [50]. In addition, plants also regulate SA metabolism to coordinate growth and development with defense processes. For example, transcriptomic analysis of the rutabaga cultivars Laurentian (susceptible) and Wilhelmsburger (resistant) inoculated with pathotype 3A indicated a comparatively stronger downregulation of SABATH family genes in Wilhelmsburger during early disease development. This downregulation suggests that plants showing resistant interactions maintain high levels of free SA by reducing the rate of SA methylation [51]. Therefore, the higher SA conjugate to free SA ratio observed during susceptible interactions and the relatively high SA conjugate to free SA ratio in response to the more virulent pathotype 3A also suggest that stronger inactivation of SA through conjugation facilitates clubroot development.

### 2.4. Roots of Susceptible Inoculated Plants Have Low JA Levels

**Figure 4:**
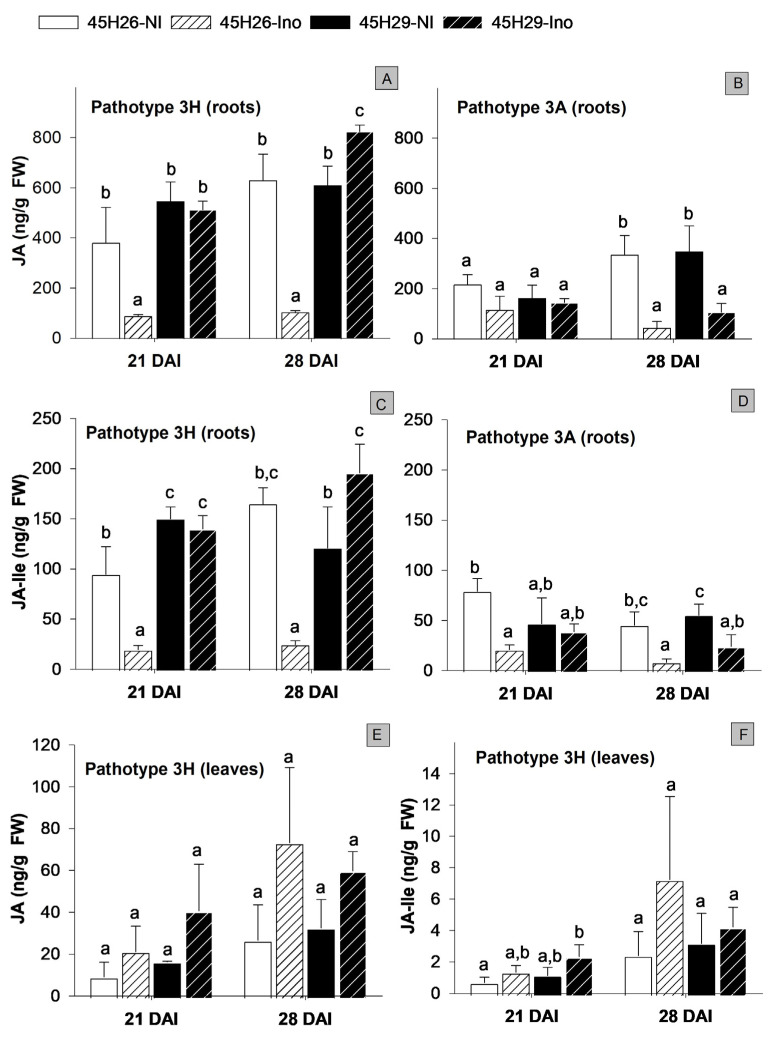
Effect of *Plasmodiophora brassicae* inoculation on jasmonic acid (JA) levels in canola root and leaf tissues at 21 and 28 days after inoculation (DAI). JA (**A**,**B**) and JA-Ile (**C**,**D**) levels in the roots of cultivars 45H26 and 45H29 inoculated (Ino) with *P. brassicae* pathotypes 3H (virulent on 45H26 and avirulent on 45H29) or 3A (virulent on both cultivars) and in non-inoculated (NI) controls. JA (**E**) and JA-Ile (**F)** levels in the leaves of 45H26 and 45H29 plants inoculated with pathotype 3H and in non-inoculated controls. Data are means ± SD, n = 3 (with each sample composed of 24–30 plants). Different letters denote significant differences within DAI as determined by one-way ANOVA followed by the Holm–Sidak post-hoc test (*p* ≤ 0.05).

In the absence of *P. brassicae* inoculation, 45H26 and 45H29 showed no cultivar-dependent difference in JA levels in the roots and leaves (21 and 28 DAI controls). Inoculation with pathotype 3H decreased JA levels in the roots of the susceptible cultivar (45H26) by about 4- and 6-fold at 21 and 28 DAI, respectively, compared with the non-inoculated controls (Figure 4A). JA levels were either the same (21 DAI) or slightly increased (28 DAI) in the resistant cultivar 45H29 when inoculated with pathotype 3H. In the leaves, there appeared to be a trend of increasing JA in response to infection by pathotype 3H, irrespective of plant susceptibility; however, those changes were not significant relative to their respective non-inoculated controls (Figure 4E). Apart from changes in JA levels in the roots, it is possible that higher SA levels in *P. brassicae*-inoculated plants may also suppress JA activity, considering the well-known antagonistic interactions between SA and JA [26,52]. Supporting this possibility, transcriptomic analysis has revealed that clubroot infection in *B. napus* leads to an increase in the transcript level of the SA-inducible transcription factor WRKY70, which acts as a key regulator of the SA-JA antagonism and a negative regulator of JA action [43,53].

Plants convert JA into multiple derivatives, including JA-isoleucine (JA-Ile), the biologically active form of JA [54]. The JA-Ile level in the roots of pathotype 3H-inoculated plants showed a trend similar to that observed with JA, with approximately 5- and 7-fold declines in the susceptible 45H26 at 21 and 28 DAI, respectively. In the resistant cultivar 45H29, the JA-Ile levels did not change in response to inoculation with pathotype 3H at 21 DAI but increased slightly at 28 DAI (Figure 4C). No significant difference in JA-Ile levels was detected in the leaves, irrespective of the susceptibility to pathotype 3H (Figure 4F).

Plants inoculated with pathotype 3A, which is virulent on both cultivars, showed no significant changes in JA levels at 21 DAI (Figure 4B). However, reductions of approximately 7.5- and 3-fold were observed in 45H26 and 45H29 by 28 DAI, respectively, when compared with the respective non-inoculated controls. JA-Ile levels were also reduced in pathotype 3A-inoculated plants with approximately 4- and 5-fold reductions in 45H26 at 21 and 28 DAI, respectively, and a 2-fold reduction in 45H29 at 28 DAI (Figure 4D). These observations further suggest that infection by *P. brassicae* causes a decrease in JA levels in the roots of susceptible plants during rapid root gall enlargement. Supporting this reduction in JA in *P. brassicae*-infected plants, transcriptomic analysis in *B. napus* shows that both susceptible and resistant cultivars exhibited reduced expression of *JASMONATE RESISTANT 1* (*JAR1*), a JA-amino synthetase coding gene required for the conversion of JA to JA-Ile [55], when inoculated with *P. brassicae* [43].

In contrast to the observed reduction in JA in susceptible plants, several other studies have reported no changes or increased JA in *P. brassicae*-infected plants. The JA level increased at 14 and 17 DAI in the partially resistant Arabidopsis accession Bur-0 and susceptible accession Col-0 when inoculated with *P. brassicae* isolate eH [26]. In the *B. napus* cultivar SY Alister (partially resistant) and Hornet (susceptible) inoculated with pathotype 6 (*sensu* Williams, 1966) [42], no significant shifts in root JA levels were observed irrespective of clubroot susceptibility at most time-points from early to late disease development, except for some intermittent increases [25]. In a third study with the *B. napus* cultivar ‘Zhongshuang 11’, increased root JA levels were reported at 14 and 28 DAI, while slight reductions were reported at 3 and 4 DAI [56]. Therefore, observed changes in JA may depend on the host, pathotype and/or the stage of disease development.

Attempts to evaluate the role of JA using JA treatments have also produced inconsistent results. For example, in *B. napus*, the application of JA at 15 DAI increased gall formation both in the susceptible cultivar Hornet and the partially resistant cultivar SY Alister [25]. In contrast, in Arabidopsis, repeated applications of methyl jasmonate initiated at 10 DAI reduced disease development in the susceptible ecotype Col-0, but not in the partially resistant Bur-0 [26]. As Bur-0 has been shown to increase SA biosynthesis in response to *P. brassicae* infection [26] and SA is known to have antagonistic interactions with JA, a lack of response in Bur-0 may be associated with high SA levels. Therefore, the reductions in JA and JA-lle levels during gall development in both canola cultivars at 28 DAI, combined with the observation of stable or increased JA levels during resistant interactions in this study and other similar research, indicate that an optimal level of JA activity may support the resistance response to *P. brassicae*.

### 2.5. Auxin Level Fluctuates in the Roots of Susceptible Inoculated Plants

**Figure 5:**
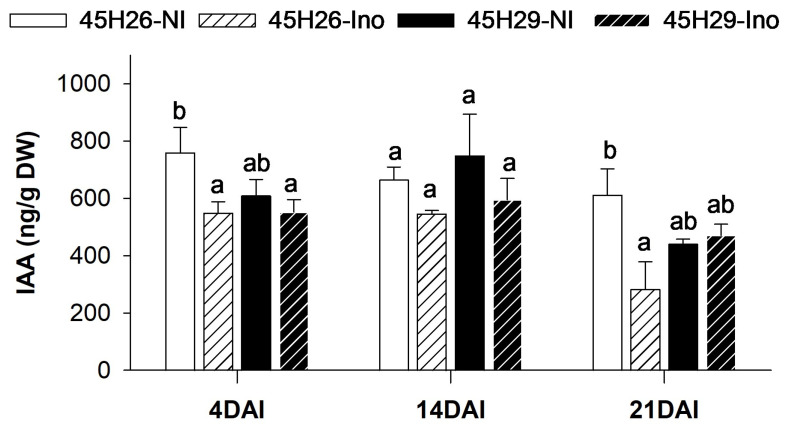
Modulation of root auxin (IAA) level in response to clubroot disease development. Levels of IAA in the canola cultivars 45H26 (susceptible) and 45H29 (resistant) inoculated (Ino) with *Plasmodiophora brassicae* pathotype 3H and in respective non-inoculated (NI) controls at 4, 14, and 21 days after inoculation (DAI). Data are means ± SD, n = 3 (with each sample composed of 60–78 plants). Different letters denote significant differences within DAI by one-way ANOVA followed by the Holm–Sidak post-hoc test (*p* ≤ 0.05).

As auxin is considered to play an important role in root gall development, changes in the root IAA profile were quantified at 4, 14, and 21 DAI. Earlier stages of disease development were selected for this analysis in order to examine shifts in the IAA profile occurring prior to gall formation. Non-inoculated plants of 45H26 and 45H29 showed no difference in IAA levels between the cultivars at any of the time-points tested (Figure 5). When inoculated with pathotype 3H, the free IAA level in the roots of the susceptible cultivar 45H26 was reduced at 4 and 21 DAI but showed no change at 14 DAI. In contrast, no significant differences were detected in the resistant cv. 45H29 at any of the time-points tested. These data suggest that modulation of auxin homeostasis was associated with host susceptibility to clubroot.

Apart from free IAA, we also analyzed its amino acid conjugates IAA-Ala, AA-Asp, IAA-Glu, and IAA-Leu. The levels of IAA-Ala, IAA-Asp and IAA-Leu were below the sensitivity range of our analysis. In contrast, IAA-Glu was detected consistently in the plant roots at 4 DAI, but its levels varied widely across replications (Table S1). While this suggests that IAA-Glu might be a prominent auxin amino acid conjugate in the roots of young canola plants, its levels showed no apparent shift in response to pathogen infection.

Several studies conducted with Brassicas have reported no change or increased IAA levels at specific time-points after *P. brassicae* infection [25,57,58,59]. In agreement with our observations, however, other studies have reported a decline in IAA. For example, the *B. napus* cv. Danestone showed reduced IAA levels when tested over multiple time-points during gall development [60]. In the Chinese cabbage cv. Wong Bok, the roots of infected plants had approximately 70% lower IAA levels than non-inoculated controls at 21 DAI, but no changes were observed at 6 or 13 DAI [29]. In another study with Chinese cabbage, both susceptible (Granat and Nippon) and tolerant (Parkin and Chorus) host genotypes showed an increase in IAA levels at 10 DAI when grown in a liquid medium containing *P. brassicae*. In contrast, IAA levels declined in the susceptible genotypes at 14 DAI, while they continued to increase in the resistant hosts [61]. From our data on IAA levels and those from other studies, it is apparent that the homeostatic mechanisms regulating free IAA levels complicate the interpretation of the role of IAA in clubroot development. A broader analysis of auxin levels, signaling and responses is required to evaluate the role of auxins in this process.

### 2.6. ACC Level Increases in Response to Pathogen Infection

**Figure 6:**
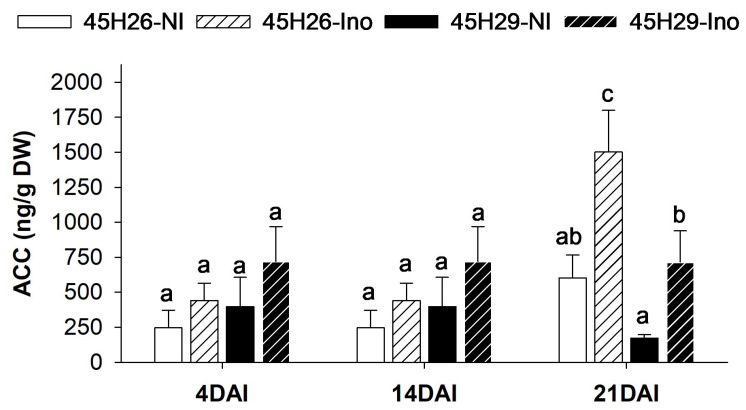
Modulation of ethylene precursor ACC level in the roots in response to clubroot disease development. Level of ethylene precursor 1-aminocyclopropane-1-carboxylic acid (ACC) in the canola cultivars 45H26 (susceptible) and 45H29 (resistant) inoculated (Ino) with *Plasmodiophora brassicae* pathotype 3H and in respective non-inoculated (NI) controls at 4, 14, and 21 days after inoculation (DAI). Data are means ± SD, n = 3 (with each sample composed of 60–78 plants). Different letters denote significant differences within DAI by one-way ANOVA followed by the Holm–Sidak post-hoc test (*p* ≤ 0.05).

As a gaseous hormone that stimulates plant responses at very low levels, ethylene is particularly challenging to monitor in root tissues. The level of the immediate ethylene precursor 1-aminocyclopropane-1-carboxylic acid (ACC) is commonly measured as an estimator of potential ethylene production, as biosynthesis of ACC generally acts as the rate-limiting step of ethylene production [62]. In this study, ACC levels were quantified in the same samples used for auxin analysis. When non-inoculated 45H26 and 45H29 plants were compared at 4, 14 and 21 DAI, the two cultivars showed no differences in root ACC level. Inoculation with pathotype 3H also had no effect on ACC levels in either cultivar at the two early time-points, but both showed an increase in ACC at 21 DAI. While inoculated susceptible plants (45H26) had the highest ACC level overall, the increase was approximately 2.5-fold in susceptible plants and 4-fold in resistant plants compared with their respective non-inoculated controls (Figure 6).

The increase in ACC levels in 3H-inoculated plants of both the resistant and susceptible cultivars suggests that this increase could be associated with plant defense responses rather than directly with root gall development and associated abiotic stress responses. However, the roots of *P. brassicae*-infected Arabidopsis plants showed no significant change in ACC levels during disease progression, except for a reduction at 20 DAI [28]. Moreover, the ACC levels were not affected in response to clubroot development in Chinese cabbage, despite a transient increase in non-inoculated controls at 13 DAI [29]. While these observations from different species do not align completely, they indicate a potential shift in ethylene biosynthesis, at least at selected time-points, hinting at a contribution of ethylene in host–pathogen interactions. Consistent with these observations, and indicating a potential positive role in clubroot defense, the ethylene-overproducing Arabidopsis mutant *eto2* has shown a slight reduction in disease susceptibility, while the ethylene signaling mutants *etr1-1*, *ein2* and *ein3-1* have shown an increased disease susceptibility [28]. Furthermore, transcriptomic analysis conducted on the rutabaga cultivars Laurentian (susceptible) and Wilhelmsburger (resistant) inoculated with *P. brassicae* pathotype 3A revealed an increased expression of *ETHYLENE RESPONSE FACTORS* (*ERFs*) [51]. These ERFs function at the end of the ethylene signaling pathway to regulate the expression of genes responsive to ethylene. During the early stages of clubroot development (4 and 14 DAI), Wilhelmsburger exhibited a greater number of upregulated transcripts annotated as ERFs, suggesting a potential positive role of ethylene in disease resistance [51]. However, in another study involving susceptible and resistant *B. napus* cultivars inoculated with pathotype 5X, it was found that *ERF4* and *ERF2* were mostly downregulated in both cultivars [43], indicating differential expression patterns in some ethylene-responsive genes.

We also analyzed gibberellin and cytokinin levels in the roots of pathotype 3H-inoculated and non-inoculated plants at 4, 14, and 21 DAI of both cultivars. The levels of these hormones, however, were either low with no discernible pattern with respect to treatments, or not detected (Tables S2 and S3). The cytokinin riboside cZR was an exception, but its level showed no difference in response to canola cultivar or inoculation status (Tables S2 and S3).

## 3. Materials and Methods

### 3.1. Plant and Pathogen Material

Two *P. brassicae* pathotypes, 3A and 3H, classified according to the Canadian Clubroot Differential system [3], were used. For pathotype 3H, root galls originating from a single-spore isolate (SACAN-ss1) [63] were used. Root galls infected with pathotype 3A were obtained from a local field population. All galled root materials were kept in a −20 °C freezer until inoculum preparation. The canola cultivars 45H26 and 45H29 (Corteva Agriscience, Saskatoon, SK, Canada) were used to evaluate disease development and physiological changes. The cultivar 45H26 is susceptible to both pathotypes 3A and 3H, while 45H29 is susceptible to pathotype 3A but resistant to pathotype 3H [3].

### 3.2. Growth and Inoculation of Plants

The procedures for preparing *P. brassicae* resting spore suspensions and conducting inoculations were adapted from [64]. Briefly, pre-washed gall material was ground in a blender in approximately 1000 mL of Milli-Q water per 100 g of root galls. The resulting homogenate was filtered through six layers of cheesecloth to remove plant and other debris, and the spore concentration in the filtrate was estimated with a hemocytometer and adjusted to approximately 5 × 10^7^ spores/mL using Milli-Q water. The resulting spore suspensions were used immediately or kept at 4℃ overnight before inoculation.

Canola seeds were germinated on a layer of wet filter paper in Petri plates. When inoculating for hormone analysis studies, seven-day-old seedlings were dipped in the spore suspension for about 10 s. Each seedling was planted in standard garden tray inserts containing Sunshine LA4 potting mixture (Sunshine Growers, Vancouver, BC, Canada). After planting, 500 μL of the spore suspension was added to the potting mix to ensure success of the inoculation. When inoculating canola for all the other studies, seven-day-old seedlings were first planted on the soil, and 2 mL of the spore suspension was added directly to the potting mix around the seedlings. Non-inoculated control plants were treated with distilled water. The plants were maintained in a greenhouse with an average temperature of 23 °C and a 16 h photoperiod. When two different cultivars were used for a particular study, each cultivar was grown in separate trays. In addition, the inoculated and non-inoculated plants were always kept and handled separately to avoid any cross-contamination with the pathogen. Plants were kept saturated with water for the first 4 DAI and watered as required thereafter, except in the water absorption study described below. From the second week onwards, plants were fertigated with 20-20-20 NPK weekly.

### 3.3. Disease Rating

Clubroot disease rating was conducted at 42 DAI as described in [64] using a 0–3 scale, where 0 = no root galls, 1 = only minor root galls, most roots are symptom-free, 2 = moderate galls, some roots are symptom-free, and 3 = larger galls, no symptom-free roots. An index of disease (ID) was calculated using the following formula: ID (%) = {[∑(0 × *n* + 1 × *n* + 2 × *n* + 3 × *n*)]/*N* × 3} × 100, where 0–3 = ratings in the disease severity scale, *n* = number of plants in each rating, and *N* = total number of plants. The experiment consisted of 4 treatments (2 applications, inoculated and non-inoculated × 2 cultivars) with 3 replicates per treatment. Each replicate consisted of one 36-cell insert tray block (each cell in the tray measured 4 cm × 4 cm × 5.5 cm) with one plant per cell (36 plants in total, per replication per treatment). The replicates were arranged in a completely randomized design.

### 3.4. Water Absorption Study

Plants of the cultivar 45H26 were used for the water absorption study. The study consisted of 2 treatments (with and without inoculation with pathotype 3H) with 3 replicates per treatment. Each replicate consisted of one 48-cell insert tray block (6.5 cm × 4 cm × 5.7 cm per cell) with 1 plant per cell (40 plants total). The replicates were arranged in a completely randomized design. When preparing plant trays, an equal amount (by weight) of potting mix was added to each tray. Each 48-cell insert tray was placed on a second, larger plastic tray filled with water to maintain water-saturated conditions. At the end of the first week, all of the remaining water was removed. The eight cells without plants at a corner of each insert tray were also removed so that water could be poured directly into the plastic tray holding the insert trays, and the trays were filled with an exact amount (2–3 L) of water every 2–3 days. The water remaining in each tray was measured and discarded 2–3 h after watering. Liquid fertilizer was applied once a week following the same approach. The plant water demand was calculated as a percentage of the average amount of water absorbed by the control plants at each time-point. The study was repeated a second time with similar results.

### 3.5. Chlorophyll Analysis

For chlorophyll analysis, the study consisted of 20 treatments (2 cultivars, 45H26 and 45H29 × 2 applications, inoculated or non-inoculated with pathotype 3H × 5 time-points, 14, 21, 28, 35, 42 DAI). Plants were grown in the 72-cell trays (each cell in the tray measured 4 cm × 4 cm × 5.5 cm) with one plant per cell (3 trays per cultivar for non-inoculated treatments and 3 trays per cultivar for inoculated treatments). Leaf tissue samples were collected from nine plants per cultivar per treatment at each time-point (three plants were randomly collected from each tray) and each plant was considered as a replication. Two leaf discs were excised from one mature leaf per plant with a 7.5 mm diameter (#3) cork-borer from the same symmetrical positions adjacent to the midrib. As mature leaves are most likely to show a loss of chlorophyll in response to disease progression, leaves towards the base of the plants were selected for this analysis. However, the most mature leaf at the lowest position of the plants, which may show natural loss of chlorophyll irrespective of disease development, was avoided. Accordingly, at 14 and 21 DAI, discs were collected from leaves attached to the second node from the bottom (excluding the node, to which cotyledonary leaves were attached). As the plants lost mature leaves with development, leaves attached to the third node were collected at 28 DAI. At 35 and 42 DAI, the samples were collected from the second remaining leaf from the bottom, irrespective of the node position. Leaf discs were harvested onto ice and stored at −80 °C until analysis.

For chlorophyll extractions, 4 mL of dimethyl sulfoxide (DMSO) was added to each pair of leaf discs and incubated at 65 °C for 90 min, with gentle agitation every 30 min. Approximately 1.5 mL of chlorophyll extract was placed in a disposable acrylic cuvette, and absorbance was measured at wavelengths of 649 nm and 665 nm with a NanoDrop 2000c (Thermo Fisher Scientific, Waltham, MA, USA) spectrophotometer. Chlorophyll *a* and *b* (*C_a_* and *C_b_*) concentrations were estimated according to the equations below as described by Wellburn, 1994 [65] for spectrophotometers with a resolution range of 1–4 nm.

$$C_{a}=12.19\;A_{665}-3.45\;A_{649}\;C_{b}=21.99\;A_{649}-5.32A_{665}$$

### 3.6. Photosynthetic Measurements

Clubroot-susceptible seedlings of the cultivar 45H26 were used in this study. The experiment consisted of 8 treatments (2 applications, non-inoculated or inoculated with pathotype 3H × 2 time-points, 21 and 28 DAI × 2 leaf positions, leaf 3 and leaf 4). Each treatment had 12 replications, with individual plants considered as replications. Seedlings were planted in the 48-cell insert trays with 42 plants per tray. Separate trays were prepared per inoculation type per time-point. The plants were grown in the greenhouse in 47.5 cm × 47.5 cm × 93.0 cm insect-resistant cages (BugDorm cages, MegaView Science, Taiwan) to avoid any insect interactions. On the day before the photosynthetic measurements at 21 and 28 DAI were to be made, one tray of inoculated plants and one of non-inoculated controls were moved to the laboratory, and 12 plants were randomly selected. Measurements were taken on the third and fourth true leaves in the laboratory environment with a CIRAS-3 Portable Photosynthesis System attached to a PLC3 Universal Leaf Cuvette containing an 18 mm diameter window (PP Systems, Amesbury, MA, USA). The system was used to analyze net photosynthesis, stomatal conductance, transpiration, and photosynthetic water use efficiency. The CO_2_ concentration, air temperature, and photosynthetically active radiation (PAR) in the cuvette were maintained at 390 ppm, 25 °C, and 1000 μmol m^−2^ s^−1^, respectively.

### 3.7. Hormone Profiling

Hormone profiling for IAA, ACC, and ABA was conducted at 4, 14, and 21 DAI. These time-points were selected based on our hypothesis that IAA plays a larger role in early disease development, given its involvement in cell division and enlargement. Additionally, we considered the possibility of a significant shift in ABA levels due to the distinct changes in the extent and severity of root galling over this period, which could affect plant water uptake, influencing shifts in ABA levels. ACC analysis was also performed at these time-points, given the possibility of simultaneously performing analysis using the same samples. For SA and JA, hormone profiling was conducted at 21 and 28 DAI, as these time-points coincide with the presence of large, actively developing root galls. We anticipated clear differences in defense hormone levels between resistant and susceptible plants at this stage due to contrasting pathogen loads.

The experiment to obtain IAA, ACC, and ABA profiles in the root tissue consisted of 12 treatments (2 applications, non-inoculated and inoculated with pathotype 3H × 2 cultivars, 45H26 and 45H29 × 3 time-points, 4, 14, 21 DAI). There were three replicates (samples) per treatment. Plants were grown in 72 cell trays (each cell in the trays measured 4 cm × 4 cm × 5.5 cm), with 1 to 1.5 trays per replication. Root tissues were harvested from 60–78 plants per treatment per replication.

For obtaining SA and JA profiles in the root tissues, the experiment consisted of 16 treatments (2 applications, non-inoculated and inoculated × 2 pathotypes, 3H and 3A × 2 cultivars, 45H26 and 45H29 × 2 time-points, 21 and 28 DAI). There were three replications per treatment. Plants were grown in 72 cell trays as above with 0.5 to 1 tray per replication. Root samples were collected from 36 to 54 plants per treatment per replication.

For obtaining SA and JA profiles in the leaf tissues, the experiment consisted of eight treatments (2 applications, non-inoculated and inoculated with pathotype 3H × 2 cultivars, 45H26 and 45H29 × 2 time-points, 21 and 28 DAI), with three replications per treatment. The same set of plants used for SA and JA analysis in the root tissues with pathotype 3H was used for leaf sample collections, but only 24–30 plants were randomly chosen per treatment per replicate.

All the plant trays were kept inside insect cages as described above to avoid any insect interactions. Roots were washed thoroughly with DI water, and trapped debris was removed with a tweezer. Cleaned roots were excised from the plants, blot-dried, flash-frozen in liquid nitrogen and stored at −80 °C. When harvesting leaves, all the fully opened true leaves, except the first true leaf and those with any visual damage, were collected (average of three leaves per plant) by clipping at the leaf base, frozen immediately in liquid nitrogen and stored at −80 °C.

All the hormone profiling was conducted at the National Research Council (NRC), Saskatoon, Canada, on a UPLC/ESI-MS/MS utilizing a Waters ACQUITY UPLC system equipped with a binary solvent delivery manager and a sample manager coupled to a Waters Micromass Quattro Premier XE quadrupole tandem mass spectrometer via a Z-spray interface. MassLynx™ and QuanLynx™ (Micromass, Manchester, UK) were used for data acquisition and analysis. The quantification of SA and JA was performed using a modified procedure described in [41,66], while the acidic hydrolysis of conjugated SA was carried out through a modified procedure described in [41]. The protocol for quantifying ABA and ABA catabolites, cytokinins, auxin, and gibberellins was modified from [67]. The quantification of ACC was performed using a modified procedure described in [68]. Briefly, the analyses utilized the Multiple Reaction Monitoring (MRM) function of the MassLynx v4.1 (Waters Corporation, Milford, MA, USA) control software. The resulting chromatographic traces were quantified off-line with QuanLynx v4.1 (Waters Corporation, Milford, MA, USA), wherein each trace was integrated and the resulting ratio of signals (nondeuterated/internal standard) was compared with a previously constructed calibration curve. Calibration curves were generated from the MRM signals obtained from standard solutions based on the ratio of the chromatographic peak area for each analyte to that of the corresponding internal standard (Supplementary Protocol S1 for internal standards used in the analysis) [69,70,71]. The QC samples, internal standard blanks, and solvent blanks were also prepared and analyzed along with each batch of tissue samples.

### 3.8. Statistical Analysis

Statistical analysis was carried out with SigmaPlot 13 (Systat Software Inc., San Jose, CA, USA). Significant differences between treatments and/or cultivars were analyzed using one-way ANOVA followed by a Holm–Sidak post-hoc mean separation test. The only exception was when comparing leaf chlorophyll levels between inoculated and non-inoculated plants, where comparisons were made using the two-tailed Student’s t-test. The data means are the average of biological replicates (samples), and the number of samples is given within the relevant data set. Statistical significance was declared at *p* ≤ 0.05. Error bars in the graphs represent the standard deviation when the number of samples is ≤4 or standard error when the number of samples is >4.

## 4. Conclusions

**Figure 7:**
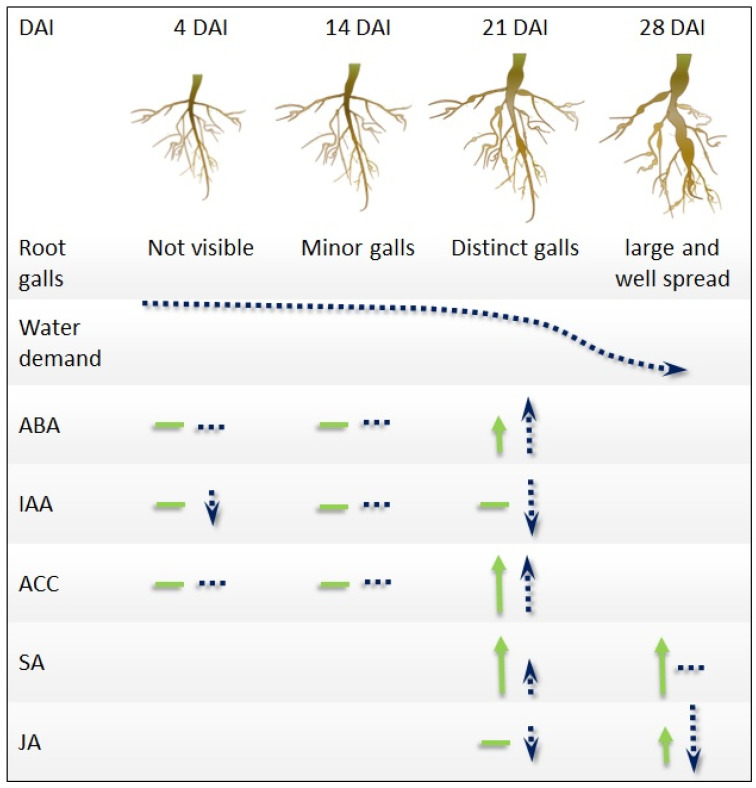
A time-course representation of clubroot disease development and associated changes in hormonal profiles in susceptible and resistant interactions between *Plasmodiophora brassicae* and canola. Seven-day-old canola plants infected with *P. brassicae* develop minor galls by 14 days after inoculation (DAI). The galls become easily recognizable by 21 DAI, and by 28 DAI, they have enlarged and spread into most roots. Plant water uptake declines parallel to gall formation, with a sharp decline starting around 21 DAI. Infected plants show an increased ABA level with declining water uptake, with susceptible plants showing a larger increase. The level of IAA in the roots in susceptible inoculated plants declines early following inoculation and during gall enlargement. Irrespective of the plant susceptibility, the level of the ethylene precursor ACC increases in infected plants by 21 DAI. SA levels primarily increase in response to resistant interactions, while JA levels decline only during susceptible interactions. The horizontal lines and arrows indicate no or limited changes and increases or decreases, respectively, compared with non-inoculated controls. Resistant interactions are in solid green and susceptible interactions are in dotted blue. Shorter arrows show relatively smaller shifts in hormonal profiles compared with the other interaction type or time-points.

Our study provides insights into the complex contribution of plant hormones to clubroot development and the maintenance of physiological functions in canola plants infected with *P. brassicae*. The visual appearance of distinctive root galls in *B. napus* around 3–4 weeks following inoculation is likely causing a major shift in plant physiology, as observed by the rapid decline in water demand and changes in hormonal profiles. Almost all plant hormonal profiles studied were impacted during this period, with ABA, IAA, and JA mainly affected by susceptible interactions, SA by resistant interactions, and ethylene by both susceptible and resistant interactions (Figure 7). Increased ABA levels in susceptible plants is likely to be mainly due to clubroot-induced stress including interference with water uptake. The declines in JA and IAA in roots only during susceptible interactions also suggest a potential connection of those hormones to physiological responses associated with pathogen infection and gall development. Higher SA levels observed during resistant compared with susceptible interactions support previous observations that SA is likely a key player in plant defense against *P. brassicae*. The pathogen’s ability to overcome SA-mediated plant defense in susceptible plants could be due to the pathogen-mediated suppression of SA availability and a reduced capacity of those plants to trigger SA-mediated defense responses. Moreover, the significant increase in ABA levels in susceptible plants, coupled with evidence from other pathosystems, indicating a potential antagonistic effect of ABA on SA action, suggests that high ABA levels in susceptible plants may also suppress SA-mediated defense mechanisms, thereby facilitating disease development. In addition, increased ACC levels in the roots of infected plants also support a contribution of ethylene in plant–pathogen interactions, regardless of host susceptibility.

The activation of plant defense responses, physiological processes and responses associated with root gall development, interactions among different plant hormones, and potential pathogen-mediated modulations of hormonal biosynthesis and metabolism can all be contributing factors to the changes observed in hormonal profiles. While our observations aligned well with some previous studies, others have reported conflicting observations, making it difficult to predict a common model describing hormonal action during clubroot development. The discrepancies in observations could be due to multiple reasons: environmental factors such as plant growth conditions and soil or potting mix properties, differences in pathotype virulence, viability and inoculum density, and species and cultivar-specific plant responses. In this study, we have taken the initial steps to explore the impact of different pathotypes and canola cultivar combinations on hormone profiles. Our data suggest that the changes in hormonal profiles are at least partially dependent on the type of interaction between the pathotype and cultivar. The next step in developing a hormonal regulatory model would be to compare changes in hormonal profiles among different host species. A challenge here is the difficulty of translating observations made in different species to a common timeframe, as the rate of disease progression is species-dependent. Therefore, synchronization of clubroot developmental timelines in different Brassica species is required to compare hormonal profiles between host species. Such an approach would eventually help to resolve discrepancies and understand the possibility of modulating plant hormonal profiles as a tool for clubroot defense. However, to validate the plant hormonal roles in clubroot disease development, including the ones described in our study, further analysis is essential beyond hormonal profiling. This entails conducting genetic and biochemical examinations, including the evaluation of hormonal biosynthesis and signaling mutants.

## Data Availability

The data presented in the study are included in the article/Supplementary Material. Further inquiries can be directed to the corresponding authors.

## Supplementary Materials

The following supporting information can be downloaded at: https://www.mdpi.com/article/10.3390/plants12162899/s1, Figure S1: Stomatal conductance to water vapor, transpiration rate, and photosynthetic water use efficiency in plants of the canola cv. 45H26 inoculated or non-inoculated with *Plasmodiophora brassicae* pathotype 3H; Figure S2: The ratio of conjugated salicylic acid to free SA in the roots and leaves of the canola cultivars 45H26 and 45H29 at 21 and 28 days after inoculation with *Plasmodiophora brassicae*; Figure S3: The ratio of conjugated salicylic acid to free SA in the roots of the canola cultivars 45H26 and 45H29 at 21 and 28 days after inoculation with *Plasmodiophora brassicae;* Table S1: IAA-glutamate in the roots of the canola cultivars 45H26 and 45H29 inoculated with *Plasmodiophora brassicae* pathotype 3H and in non-inoculated controls; Table S2: GA_3_ and GA_8_ in the roots of the canola cultivars 45H26 and 45H29 inoculated with *Plasmodiophora brassicae* pathotype 3H and in non-inoculated controls; Table S3: Cytokinin levels in the roots of the canola cultivars 45H26 and 45H29 inoculated with *Plasmodiophora brassicae* pathotype 3H and in non-inoculated controls; Protocol S1: Standards used in the UPLC/ESI-MS/MS quantification of plant hormones.
