# Supplementary material for: Impact of Susceptibility on Plant Hormonal Composition during Clubroot Disease Development in Canola (Brassica napus)
Source: Plants (Basel). 2023 Aug 9;12(16):2899. doi: 10.3390/plants12162899 (PMC10459861; doi:10.3390/plants12162899)
Supplement: Supplementary file 1 [file plants-12-02899-s001.zip › plants-2528286-supplementary.pdf]

Supplementary Material

Impact of susceptibility on plant hormonal composition during clubroot disease development in canola (*Brassica napus*)

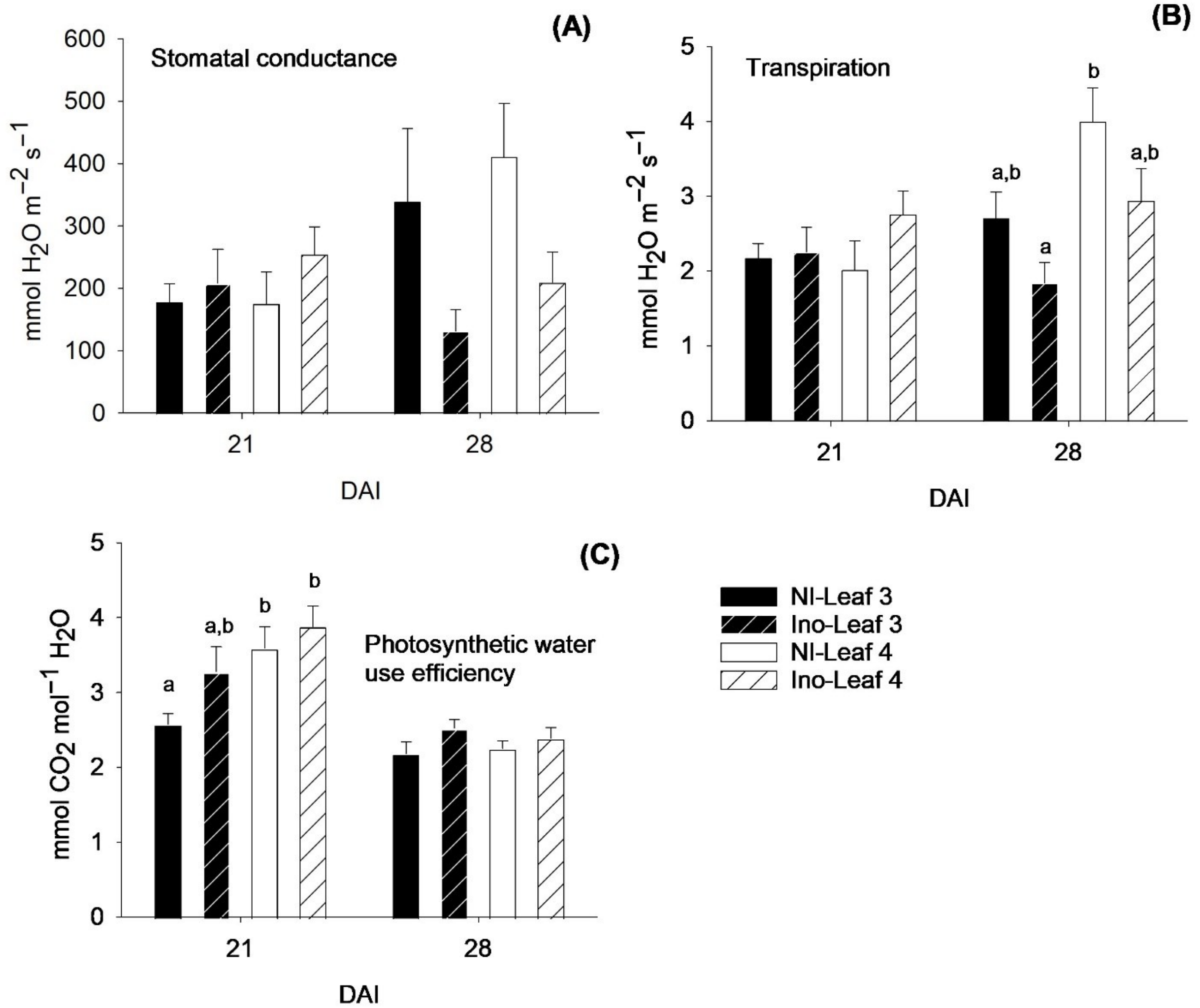

Supplementary Fig. S1 Stomatal conductance to water vapor (**A**), transpiration rate (**B**), and photosynthetic water use efficiency (**C**) in plants of the canola cv. 45H26 (clubroot-susceptible) inoculated (Ino) or non-inoculated (NI) with *Plasmodiophora brassicae* pathotype 3H. Measurements were taken from the 3rd, and 4th true leaves of plants. Data are means ± SD. Different letters denote significant differences within days after inoculation (DAI; One-way ANOVA, Holm-Sidak post-hoc test, $P < 0.05$).

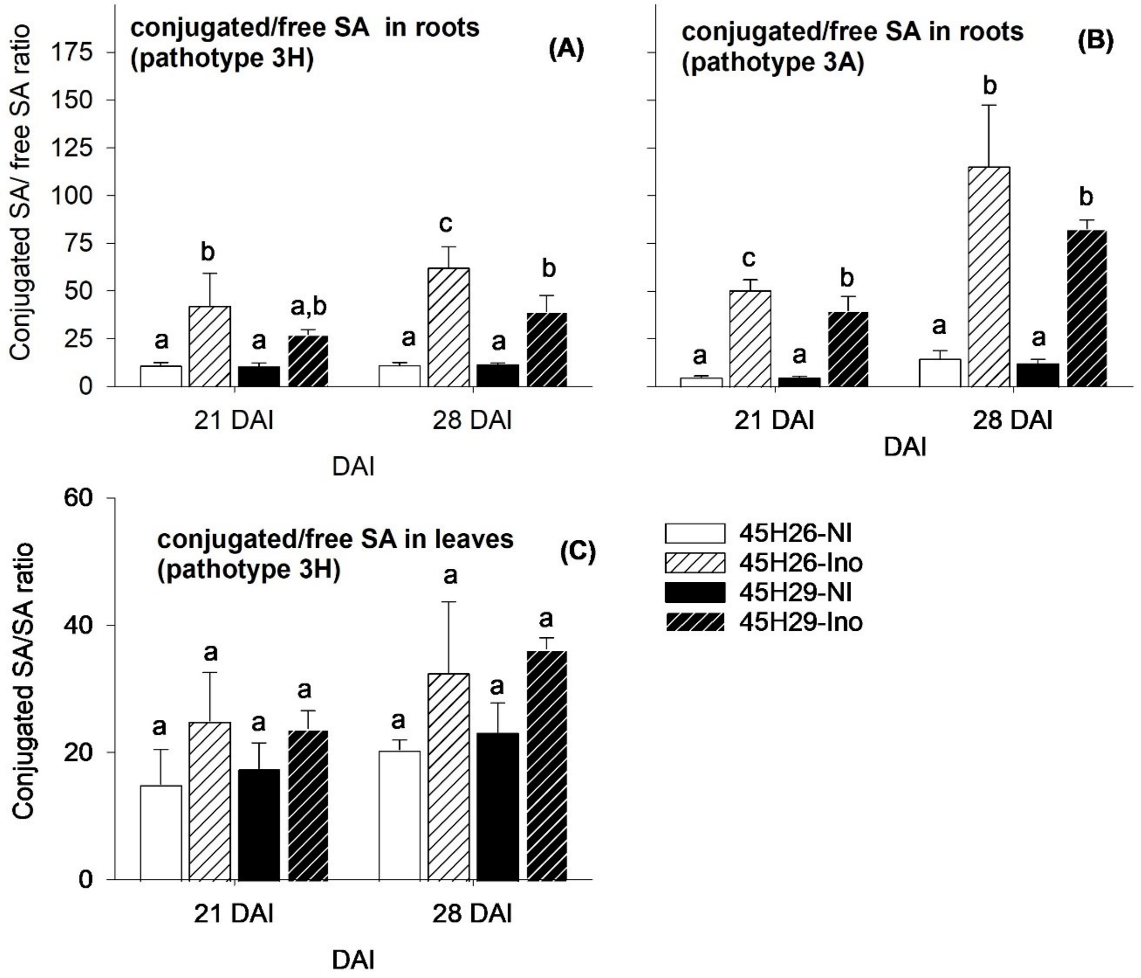

Supplementary Fig. S2. The ratio of conjugated salicylic acid (SA) to free SA in the roots (A, B) and leaves (C) of the canola cultivars 45H26 (susceptible) and 45H29 (resistant) at 21 and 28 days after inoculation (DAI) with *Plasmodiophora brassicae*. The plants were inoculated (Ino) with *P. brassicae* pathotype 3H, 3A or kept as non-inoculated controls (NI). Data are means ± SD (n=3). Different letters denote significant differences within the DAI as determined by one-way ANOVA followed by the Holm-Sidak post-hoc test ($P \leq 0.05$).

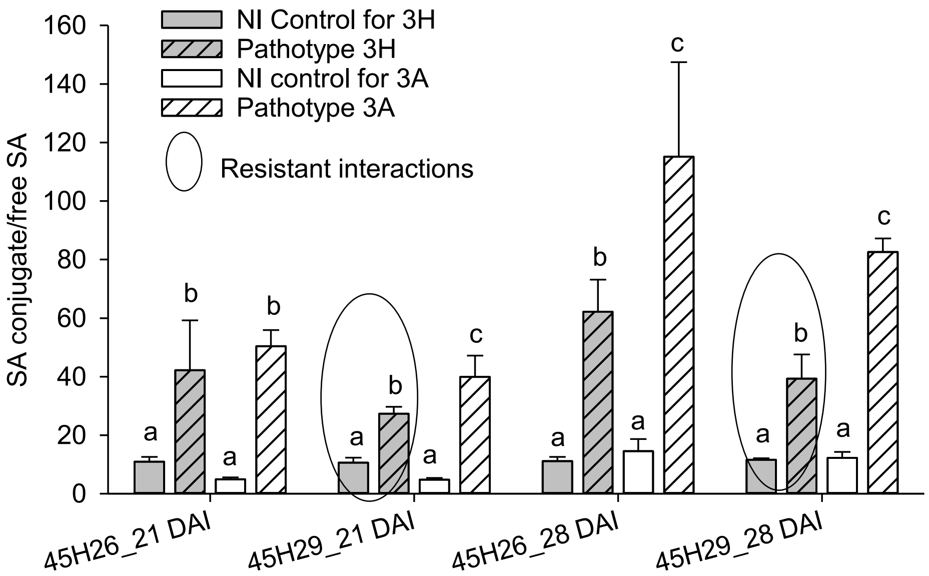

Supplementary Fig. S3. The ratio of conjugated salicylic acid (SA) to free SA in the roots of the canola cultivars 45H26 (susceptible) and 45H29 (resistant) at 21 and 28 days after inoculation (DAI) with *Plasmodiophora brassicae*. The plants were inoculated with *P. brassicae* pathotype 3A, 3H or kept as non-inoculated controls (NI). Data are means ± SD (n=3). Different letters denote significant differences within the DAI and cultivar as determined by one-way ANOVA followed by the Holm-Sidak post-hoc test ($P \leq 0.05$).

Supplementary Table S1. IAA-glutamate (Glu) in the roots of the canola cultivars 45H26 (susceptible) and 45H29 (resistant) inoculated (Ino) with *Plasmodiophora brassicae* pathotype 3H and in non-inoculated controls (NI).

**DAI** [a]	**Replicate**	**45H26**		**45H29**	
		**NI** [b]	**Ino**	**NI**	**Ino**
**4**	**1**	64.0	208.8	150.8	12.7
	**2**	n.d.	10.4	402.2	39.5
	**3**	320.4	78.3	9.0	36.0
**14**	**1**	9.7	n.d.	n.d.	n.d.
	**2**	12.4	42.3	n.d.	n.d.
	**3**	n.d.	174.8	<4.2	n.d.
**21**	**1**	n.d.	12.7	10.2	n.d.
	**2**	13.2	<3.9	n.d.	n.d.
	**3**	42.5	n.d.	n.d.	16.7

[a] DAI, days after inoculation

[b] Results are expressed in nanograms per gram of dry weight. The values with < symbol means that the signals were below the limit of quantification (LOQ defined as signal/noise ratio equal to 8), and the value of LOQ is reported. ND stands for not detected, in which values were below the limit of detection (LOD defined as signal/noise ratio equal to 3).

Supplementary Table S2. GA_3 and GA_8 in the roots of the canola cultivars 45H26 (susceptible) and 45H29 (resistant) inoculated with *Plasmodiophora brassicae* pathotype 3H (Ino) and in non-inoculated (NI) controls.

GA	DAI [a]	Replicate	Susceptible (45H26)		Resistant (45H29)	
			NI [b]	Ino	NI	Ino
GA3	4	1	<4.7	5.6	<5.1	<4.3
		2	<6	<6.5	<6.1	9.0
		3	6.1	<4.3	<6.1	<4.6
	14	1	<4.4	5.3	<4.4	<4.4
		2	n.d.	<4.3	<4.7	<4.2
		3	<5.1	<4.5	<4.2	<4.1
	21	1	n.d.	5.8	<4.1	<4.3
		2	n.d.	<3.9	<3.9	<3.9
		3	<4	n.d.	5.4	<4
GA8	4	1	<5.9	<4.9	<9.2	10.9
		2	<6	<6.5	<6.1	5.7
		3	<6	<7.5	6.9	5.3
	14	1	<5.7	<4.3	<4.4	11.1
		2	17.9	7.8	13.5	5.8
		3	17.6	4.5	14.7	6.1
	21	1	<4.2	n.d.	<4.1	n.d.
		2	5.5	4.7	<3.9	4.4
		3	<4	n.d.	<4	<4

[a] DAI, days after inoculation

[b] Results are expressed in nanograms per gram of dry weight. The values with < sign mean that the signals were below the limit of quantification (LOQ defined as signal/noise ratio equal to 8), and the value of LOQ is reported. ND stands for not detected, in which values were below the limit of detection (LOD defined as signal/noise ratio equal to 3).

Supplementary Table S3. Cytokinin levels in the roots of the canola cultivars 45H26 (susceptible) and 45H29 (resistant) inoculated with *Plasmodiophora brassicae* pathotype 3H (Ino) and in non-inoculated (NI) controls.

Cytokinin compound	DAI [a]	Replicate	Susceptible		Resistant	
			NI[b]	Ino	NI	Ino
c-ZR	4	1	4.4	3.9	5.4	6.4
		2	36.9	18.1	4.5	4.6
		3	4.0	18.3	3.2	3.3
	14	1	8.0	8.9	6.8	6.2
		2	6.6	3.4	7.8	3.0
		3	10.3	7.5	3.3	4.3
	21	1	3.0	4.2	2.7	3.5
		2	2.3	3.2	2.3	6.6
		3	2.6	2.4	4.2	2.9
iP	4	1	1.4	1.7	1.5	1.7
		2	3.0	<1.6	<1.5	<1.4
		3	2.1	<1.1	<1.5	<1.1
	14	1	1.7	<1.1	1.5	<1.2
		2	<1.2	<1.1	<1.2	<1
		3	<1.3	<1.1	1.5	<1
	21	1	<1.1	n.d.	<1	<1.1
		2	<1	<1	<1	<1
		3	<1	<1	<1	<1
iPR	4	1	1.9	n.d.	<1.3	1.3
		2	3.2	3.8	<1.5	1.9
		3	1.6	4.8	<1.5	<1.4
	14	1	4.6	n.d.	4.1	n.d.
		2	6.2	2.2	2.2	1.8
		3	6.1	2.8	1.7	1.9
	21	1	2.5	3.0	1.6	3.1
		2	2.4	1.4	2.0	6.1
		3	1.9	1.3	1.5	2.4

[a] DAI, days after inoculation

[b] Results are expressed in nanograms per gram of dry weight. The values with < sign mean that the signals were below the limit of quantification (LOQ defined as signal/noise ratio equal to 8), and the value of LOQ is reported. ND stands for not detected, in which values were below the limit of detection (LOD defined as signal/noise ratio equal to 3).

Supplementary Protocol S1. Standards used in the UPLC/ESI-MS/MS quantification of plant hormones.

SA, JA ABA, IAA-Leu, IAA-Ala, IAA-Asp, IAA, Z, ZR, iPR, iP, ACC, phenyl isothiocyanate (PITC), trifluoroacetic acid (TFA) and triethylamine were purchased from Sigma–Aldrich. GAs 1, 3, 4, 7, 8, 9, 19, 20, 24, 29, 44, and 53 were purchased from the Research School of Chemistry, Australian National University (Canberra, AU). JA-Ile was purchased from OlChemim Ltd. (Olomouc, Czech Republic).

A number of compounds, including DPA, ABA-GE, PA, 7'- OH-ABA, neoPA, trans-ABA and IAA-Glu were synthesized and prepared at the National Research Council of Canada, Saskatoon, SK, Canada; Deuterated forms of the hormones d3-DPA, d5-ABA-GE, d3-PA, d4-7'-OH-ABA, d3-neoPA, d4-ABA, d4-trans-ABA, d3-IAA-Leu, d3- IAA-Ala, d3-IAA-Asp and d3-IAA-Glu were synthesized and prepared at NRCC SK according to Abrams *et al.* (2003) and Zaharia *et al.* (2005). 2,2-d2-jasmonic acid and 12,12,12-d3-jasmonic acid isoleucine (unpublished) were synthesized and prepared at NRCC SK according to Galka *et al.* (2005). dhZ, dhZR and Z-O-Glu were purchased from OlChemim Ltd. (Olomouc, Czech Republic). The d5-IAA was purchased from Cambridge Isotope Laboratories (Andover, MA); d3-dhZ, d3-dhZR, d5-Z-O-Glu, d6-iPR and d6-iP were purchased from OlChemim Ltd.; d2-GAs 1, 3, 4, 7, 8, 9, 19, 20, 24, 29, 34, 44, 51 and 53 were purchased from the Research School of Chemistry, Australian National University.

1-amino-[2,2,3,3-d4]cylopropane- 1-carboxylic acid (d4-ACC) was purchased from CDN Isotopes (Point-Claire, QC, Canada). 3,4,5,6-d4-2-hydroxybenzoic acid was purchased from CDN isotopes (Point-Claire, QC, Canada). The d4-ACC derivative PTH-d4-ACC was used as an internal standard. The deuterated forms of selected hormones used as recovery (external) standards were prepared and synthesized at NRCC SK. Calibration curves were generated for all compounds of interest, including the ACC derivatives, phenylthiohydantoin-ACC (PTH-ACC) and PTH-d4-ACC. Quality control samples (QCs) were run along with the tissue samples.

Literature cited in the Supplementary Material

Abrams, S. R., Nelson, K., and Ambrose, S. J. (2003). Deuterated abscisic acid analogs for mass spectrometry and metabolism studies. *J. Label. Compd. Radiopharm.* 46, 273–283. doi: 10.1002/jlcr.670.

Galka, P. W., Ambrose, S. J., Ross, A. R. S., and Abrams, S. R. (2005). Syntheses of deuterated jasmonates for mass spectrometry and metabolism studies. *J. Label. Compd. Radiopharm.* 48, 797–809. doi: https://doi.org/10.1002/jlcr.994.

Zaharia, L. I., Galka, M. M., Ambrose, S. J., and Abrams, S. R. (2005). Preparation of deuterated abscisic acid metabolites for use in mass spectrometry and feeding studies. *J. Label. Compd. Radiopharm.* 48, 435–445. doi: 10.1002/jlcr.939.
